# Multiple Stressors Induce Amygdalohippocampal Volume Reduction in Adult Male Rats as Detected by Longitudinal Structural Magnetic Resonance Imaging

**DOI:** 10.1016/j.bpsgos.2024.100334

**Published:** 2024-05-15

**Authors:** Rie Ryoke, Teruo Hashimoto, Ryuta Kawashima

**Affiliations:** Department of Functional Brain Imaging, Institute of Development, Aging and Cancer, Tohoku University, Sendai, Japan

**Keywords:** Amygdala, Hippocampus, Individual variability, Magnetic resonance imaging, Posttraumatic stress disorder

## Abstract

**Background:**

Traumatic events can cause long-lasting and uncontrollable fear and anxiety. Posttraumatic stress disorder is an intractable mental disorder, and neurobiological mechanisms using animal models are expected to help development of posttraumatic stress disorder treatment. In this study, we combined multiple stress (MS) and longitudinal in vivo magnetic resonance imaging to reveal the effects of long-lasting anxiety-like behaviors on adult male rat brains.

**Methods:**

Twelve male Wistar rats (8 weeks old) were exposed to the MS of 1-mA footshocks and forced swimming, while 12 control rats were placed in a plastic cage. Contextual fear conditioning with 0.1-mA footshocks in a context different from the MS was conducted 15 days after the MS for both groups. Three retention tests were administered after 24 hours and 9 and 16 days. Two magnetic resonance imaging scans were conducted, one on the day before MS induction and one the day after the third retention test, with a 32-day interval.

**Results:**

The MS group showed greater freezing responses than the control group in all retention tests. Whole-brain voxel-based morphometry analyses revealed reduced gray matter volume in the anterior amygdalohippocampal area in MS group rats compared with control rats. These volume changes were negatively associated with freezing time in the third retention test in the MS group.

**Conclusions:**

These results suggest that individual variability in the amygdalohippocampal area may be related to long-lasting fear responses after severe stress.

## Plain Language Summary

Traumatic events can cause long-lasting and uncontrollable fear and anxiety. In this study, we combined multiple stress (MS) and longitudinal in vivo magnetic resonance imaging to reveal the effects of long-lasting anxiety-like behaviors on adult male rat brains. The MS group showed greater freezing responses than the control group in all retention tests. Brain morphometry analyses revealed reduced gray matter volume in the anterior amygdalohippocampal area in MS group rats compared with control rats. These results suggest that individual variability in the amygdalohippocampal area may be related to long-lasting fear responses after severe stress.

People feel afraid during and after traumatic situations. People with posttraumatic stress disorder (PTSD) experience stress or fear even when they are not in danger. PTSD is characterized by psychopathological symptoms, including affective symptoms, intrusive memories of trauma, avoidance of trauma-related events, hyperarousal, and negative cognition and mood (1). Symptoms may persist for several weeks to a lifetime. The most recent prevalence estimate for PTSD is 15.3%, including severe to mild disorders and disorders without functional impairment (2). Although temperamental, environmental, and genetic factors have been identified in PTSD, the neurobiological mechanisms that underlie the disorder have not been clarified yet. In contrast to human research that focuses on populations already exposed to different uncontrolled traumatic events, rodent models of PTSD are advantageous for longitudinal monitoring of PTSD development from pre- to posttrauma with controlled stressors (3,4).

Because trauma-like memories are retained for a long time, a long-term experiment with multiple time points can be suitable for assessing PTSD-like behaviors (4). In the stress-enhanced fear learning paradigm, rodents that have been pre-exposed to intense stressors show enhanced fear learning in a different context with mild stressors and fear/anxiety sensitization without a stressor (5,6). Moreover, pre-exposure to multiple stressors induces long-lasting (>30 days) fear/anxiety sensitization at multiple time points (7). However, the neural underpinnings of long-lasting PTSD-like behaviors have not been extensively investigated.

It has been suggested that the neural mechanisms of fear learning involve the amygdala and hippocampus in both humans and rodents (8,9). The amygdala, which is involved in associative learning, value encoding, and emotional responses, is particularly important in patients with PTSD. Smaller amygdala volume has been shown to be correlated with more severe PTSD symptoms in combat veteran participants (10). The hippocampus is responsible for encoding, consolidating, and retrieving episodic, contextual, and spatial memories in rats (8,11). The integration of contextual information and emotional valence required for contextual fear learning is believed to occur downstream from the hippocampus to the basolateral amygdala in rats (8). In rodents, the hippocampus exhibits stress-induced volume reduction, dendritic atrophy, pyramidal neuronal spine loss, impaired long-term potentiation, and alterations in adult neurogenesis in rodents (12,13).

Neuroimaging can provide longitudinal information on in vivo whole-brain structure and function in animals. Combining multiple longitudinal behavioral tests and neuroimaging scans can shed light on the neural mechanisms that underlie PTSD-like behaviors in rodents. However, there have been no in vivo examinations of brain morphology over time for long-term fear response–enhancing effects. Here, using multiple longitudinal tests, we examined the effects of multiple stressors on fear- and anxiety-related behaviors and brain structure in adult rats. Moreover, the relationship between long-lasting fear/anxiety-related behaviors and structural changes in the brain was examined.

## Methods and Materials

**Figure 1:**
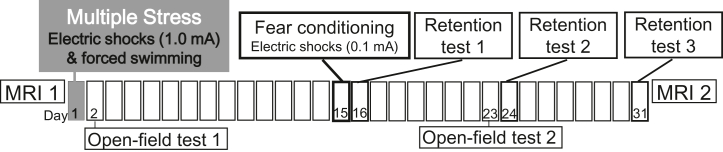
Experimental protocol. Structural MRI was performed before and after a series of behavioral tests. On day 1, the multiple stress group was exposed to multiple stressors. Both the multiple stress group and the control group engaged in the open field test on days 2 and 23 to measure anxiety-like behaviors. Both groups engaged in fear conditioning on day 15, and retention tests were conducted on days 16 (24-hour delay), 24 (9-day delay), and 31 (16-day delay). MRI, magnetic resonance imaging.

All experiments were conducted in strict accordance with the National Institutes of Health Guide for the Care and Use of Laboratory Animals and were approved by the Committee for Animal Experimentation of the Tohoku University Graduate School of Medicine (22018AcA-029). Care was taken to minimize stress and discomfort to the animals at all stages. Data were obtained from 24 male Wistar rats (8 weeks, weighing 247–315 g at the beginning of the experiment; Charles River Laboratories). The rats were housed in individual cages at 21 °C to 25 °C and maintained under a 12-hour light/dark cycle (lights on from 7 am–7 pm). Food and water were provided ad libitum. Upon arrival, rats were randomly assigned to one of the 2 experimental groups. The experimental procedures were generally consistent with those of a previous study (7), except for the magnetic resonance imaging (MRI) scans (Figure 1).

### Experimental Apparatus

#### Multiple Stress: Electric Shock Chamber

The apparatus consisted of a Plexiglas cage (length, 25 cm; width, 25 cm; height, 25 cm) with black silicon sheets covering the periphery and was placed in a draft chamber. The bottom of the chamber consisted of 27 steel rods (diameter, 3.0 mm; 5.8 mm apart) wired to a shock generator (LE10026, Panlab). Electric shocks (1 mA, 1 second, 4 times) were controlled by a stimulator (master-8; A.M.P.I.) engaged in the shock generator.

#### Multiple Stress: Forced Swimming Pool

An opaque blue plastic bucket (height, 50 cm; diameter, 40 cm) was used. For the multiple stress (MS) group, the bucket was filled with water at 25 °C to a depth of 30 cm.

### Open Field Test

The chamber was an open-top box (90 × 90 × 45 cm; O’Hara & Co., Ltd) that consisted of black polyvinyl chloride walls and a gray floor with photobeam sensors placed 20 cm above the bottom to detect vertical activities. The box was illuminated using 8 incandescent bulbs installed on the ceiling. The brightness of the center of the floor was 60 lx. A charge-coupled device camera was placed above the chamber.

### Fear Conditioning and Retention

A custom-made automated computer-controlled system was used in the habituation, conditioning, and retention test phases (14). The fear conditioning chamber (25 × 20 × 30 cm), which differed in size and background from the chamber for MS, was constructed with clear acrylic walls and included a lid with a hole in the center. For delivering electric shocks, the fear conditioning chamber was equipped with a grid floor made of 22 stainless steel rods (diameter, 3.0 mm; 8.0 mm apart) wired to a shock generator (LE10026). Electric shocks (0.1 mA, 1 second, 2 times) were controlled using a stimulator (master-8) attached to the shock generator. The chamber was located within a sound-attenuating box (70 × 50 × 65 cm; Muromachi Kikai Co., Ltd) with white inner walls and a ventilation fan that provided fresh air, background noise (50 dB), and illumination (200 lx). The animals’ behavior was monitored using 2 video cameras placed on the walls of the sound-attenuating cage.

### MS Session

On day 1, the animals in the MS group were exposed to multiple stressors. First, they were placed in a transportation box (20 × 30 × 20 cm, consisting of opaque black walls, a lid, and an opaque black smooth polypropylene floor with a black silicon sheet) for 25 minutes. Then, they experienced 4 footshocks (1 mA, 1 second, with an intershock interval varying from 4 to 6 minutes) over 25 minutes in the MS chamber. Subsequently, they were forced to swim for 20 minutes in a plastic bucket. On day 8, the MS group was exposed to a situational reminder by placing them in a transportation box for 3 minutes to enhance their memory of MS sessions.

Nonstressed (control) rats were placed in a transportation box for 25 minutes, in the MS chamber for 25 minutes without footshocks, and in a standard breeding cage for 20 minutes without water.

### Open Field Tests

The open field test is a widely used model of anxiety-like behavior developed to evaluate emotionality in animals as measured by exploratory and locomotor activities (15,16). The open field test was performed on the day after the MS session (day 2) and 22 days after the MS session (day 23). At the start of testing, each rat was placed in the left corner of the arena, and its behavior was recorded for 10 minutes. The total distance and time spent in the central and peripheral areas were recorded using the automated image analysis software TimeOFCR4 (O’Hara & Co., Ltd). In addition, the number of beam interruptions was automatically counted as the number of rearing sessions and a duration of one (standing on the hind legs).

The total distance, percentage of time spent in the central area (angular transformed values), and number of rearing sessions were compared using a 2-way repeated-measures analysis of variance (ANOVA), with group (MS vs. control) as an independent factor and delay (test 1 on day 2 and test 2 on day 23) as a repeated factor. Post hoc 1-tailed 2-sample *t* tests with Bonferroni correction for multiple comparisons were conducted. For all statistical comparisons, the significance level was set at *p* < .05. SPSS version 26 (IBM Corp.) was used to analyze the results of the open field tests, fear conditioning retention tests, and total brain volumes.

### Fear Conditioning Paradigm

#### Conditioning and Retention Test

In the habituation phase, rats were exposed to the conditioning chamber for 4 consecutive days (5 minutes/day). During the conditioning phase (day 15), each rat was placed in the conditioning chamber for 3 minutes. They were allowed to habituate for 2 minutes before receiving 2 mild footshocks (0.1 mA, 2 seconds, with an intershock interval of 1 minute). Rats were removed from the chamber immediately after the second footshock.

In the retention test phase, each rat was placed in the fear conditioning chamber again for 3 minutes without receiving shocks. Retention testing was performed on the day following the conditioning phase (day 16, 24-hour delay), day 24 (9-day delay), and day 31 (16-day delay). Fear memory strength was assessed by measuring the duration of the freezing response of the animal during the retention test. Freezing behavior, defined as the absence of any visible movement except that due to breathing, was automatically detected using homemade software and verified by an experimenter. The freezing response of animals was measured for 3 minutes. The obtained values were expressed as a percentage of the total duration of the sampled period and averaged across the trials for a given animal and among animals of the same experimental group.

Freezing amounts (angular transformed values) were compared using ANOVA, with group (MS vs. control) as an independent factor and delay (24 hours, 9 days, and 16 days) as a repeated factor, followed by post hoc 1-tailed 2-sample *t* tests with Bonferroni correction. For all statistical comparisons, the significance level was set at *p* < .05.

### MRI Data Acquisition

MRI data acquisition and analysis were generally consistent with those of our previous report that showed longitudinal brain volume changes in rats (17). Each rat was anesthetized with isoflurane (5% for initial induction and 1.5% during MRI scanning) and placed in the prone position on a Bio Animal Bed for rat’s head volume coil with a bite bar and gas mask (Bruker Biospin). Core body temperature was monitored throughout the scan using an MRI-compatible temperature probe (Model 1025; SA Instruments) inserted into the rectum and regulated at 37.0 °C ± 1 °C using a water-circulating floor heating system of the MRI bed. All MRI data were acquired using a 7T Bruker PharmaScan system (Bruker Biospin) with a 38-mm diameter birdcage coil designed to image the rat brain. T2-weighted images were obtained using the respiration-gated 2-dimensional rapid acquisition with relaxation enhancement sequence with the following parameters: repetition time, 2700 ms; echo time, 30 ms; rapid acquisition with relaxation factor, 4; field of view = 35 × 35 mm^2^; matrix size, 200 × 200; number of slices = 32; slice thickness, 1 mm; slice gap, 1 mm; number of averages, 14. The total MRI scanning time for each rat was approximately 30 minutes and was dependent on the respiration rate.

### MRI Data Preprocessing

MRI image analysis was performed using SPM8 software (Wellcome Department of Cognitive Neurology) and custom-written software in MATLAB, version R2020a (The MathWorks Inc.). First, each T2-weighted image was resized by a factor of 10 to account for the whole-brain volume differences between humans and rodents. This step was performed by varying the nominal voxel size of the image header from 0.125 × 0.125 × 0.6 mm^3^ to 1.25 × 1.25 × 6 mm^3^. Second, the resized T2-weighted image was rigid-body aligned to the stereotactic template space and resampled into 1.25-mm isotropic voxels. Third, the preprocessed T2-weighted image was segmented into probability maps of gray matter (GM), white matter (WM), and cerebrospinal fluid (CSF) using the unified segmentation approach, which enables image registration, tissue classification, and bias correction to be combined within the unified generative model. For the unified segmentation steps, the default settings in SPM8 were used except that the human tissue priors were replaced with rat tissue priors. Fourth, the tissue class images were linearly (affine transformed) and nonlinearly normalized to the stereotactic space, and the signal intensity of the normalized maps was modulated by the determinant of the Jacobian of both the linear and nonlinear components of the spatial registration to account for the expansion and/or contraction of brain regions. Finally, the resulting tissue class images (e.g., regional GM volume [rGMV]) were smoothened using a 10-mm full width at half maximum Gaussian kernel. Although image preprocessing was performed on the resized scales, the results of the voxel-based morphometry (VBM) analysis were displayed on the original scales.

### MRI Data Analyses

The total GM, WM, and CSF volumes were computed by multiplying the voxel value by the voxel volume and summing the results for all voxels. The percentage change from MRI scan 1 (MRI 1) to MRI scan 2 (MRI 2) was calculated. Statistical analyses between the MS and control groups were performed using a 2-tailed 2-sample *t* test with a significance level of .05.

Group differences in rGMV on MRI 1 and MRI 2 (each scan) were examined using 2-sample *t* tests in SPM8. The statistical significance threshold was set at *p* < .05, with familywise error correction for multiple comparisons at the cluster (extent) level and an uncorrected *p* < .001 at the voxel (height) level.

Longitudinal MR image analysis of group comparisons was performed using the pipeline described in a previous longitudinal VBM study in rats (17). To evaluate the longitudinal rGMV changes in each subject, the absolute intensity changes in the preprocessed images between MRI 1 and MRI 2 were computed at each voxel for each subject using the Image Calculator implemented in SPM8. The resulting maps (rGMV_MRI2_ − rGMV_MRI1_) were then included in a group-level analysis to investigate rGMV differences between the MS and control groups. Two-sample *t* tests across the whole brain were conducted in SPM8. The statistical significance threshold was *p* < .05, with familywise error correction for multiple comparisons at the cluster level and an uncorrected *p* < .001 at the voxel level. To further investigate individual differences in rGMV changes, we extracted the signal intensity around a 10-mm sphere of the peak voxel of the significant clusters. The relationship between rGMV changes and freezing responses in the last test of fear conditioning in the MS group was examined with Spearman’s correlation coefficients in SPSS.

## Results

### Open Field Test

**Figure 2:**
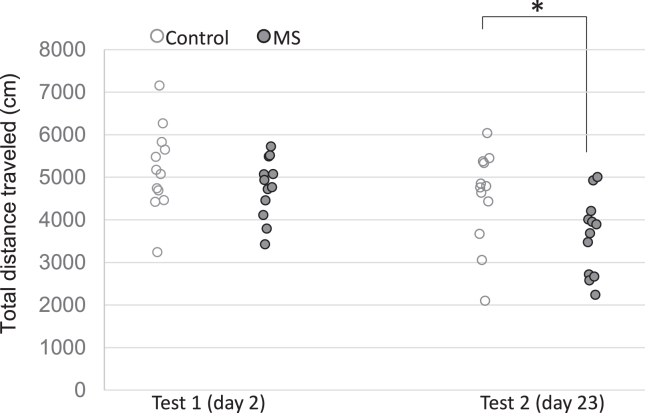
Anxiety-related reduced locomotion. Animals in the MS group showed less distance traveled than those in the control group in the open field test. While no significant group difference was observed on day 2, the MS group showed less distance traveled than the control group on day 23. ∗Significant group differences, *p* < .05. MS, multiple stress.

The mean total distance traveled was lower in the MS group than in the control group (4186 cm vs. 4862 cm) and higher on day 2 than on day 23 (4971 cm vs. 4078 cm). Two-way repeated-measures ANOVA revealed a significant effect of group (*F*_1,22_ = 4.47, *p* = .04) and delay (*F*_1,22_ = 17.32, *p* < .001) but no interaction (*F*_1,22_ = 1.37, *p* = .20). While no significant group difference was observed on day 2 (*t*_22_ = 1.19, *p* = .24), the MS group showed less distance traveled than the control group on day 23 (*t*_22_ = 2.24, *p* = .03) (Figure 2).

**Figure 3:**
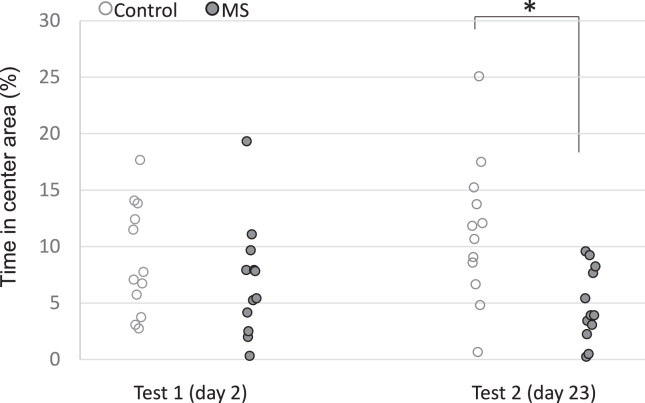
Anxiety-related exploratory behavior. Rats in the control group spent more time in the central area, showing anxiolytic and less exploratory behaviors than those in the MS group on test 2 (day 23). In contrast, the MS group showed anxiety-related exploratory behavior on test 2. ∗Significant group differences, *p* < .05. MS, multiple stress.

Mean time spent in the central area was lower in the MS group than in the control group (5.87% vs. 10.10%) and similar between day 2 and day 23 (7.90% vs. 8.06%). Two-way repeated-measures ANOVA using angular-transformed values revealed a significant effect of group (*F*_1,22_ = 17.32, *p* < .001), no significant effect of delay (*F*_1,22_ = 0.03, *p* = .84), and a significant interaction (*F*_1,22_ = 7.90, *p* = .01). No significant group difference was observed on day 2 (*t*_22_ = 0.94, *p* = .35), but the MS group spent more time in the central area than the control group on day 23 (*t*_22_ = 3.18, *p* = .004) (Figure 3).

The mean number of rearing sessions was lower in the MS group than in the control group (36 vs. 49) and was similar on days 2 and 23 (41 versus 45). Two-way repeated-measures ANOVA revealed a significant effect of group (*F*_1,22_ = 6.74, *p* = .01) but no significant effect of delay (*F*_1,22_ = 1.29, *p* = .26) or interaction (*F*_1,22_ = 2.25, *p* = .14). No significant group differences were observed on day 2 (*t*_13.37_ = 0.19, *p* = .19); however, the MS group showed more rearing sessions on average than the control group on day 23 (*t*_22_ = 2.59, *p* = .01).

### Contextual Fear Conditioning

**Figure 4:**
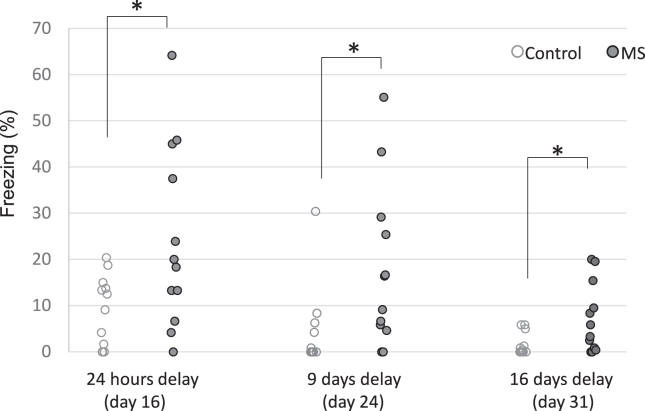
Conditioned fear responses after delays. The MS group showed exaggerated freezing compared with the control group, even after 16 days of fear conditioning. ∗Significant group difference, *p* < .05. MS, multiple stress.

The MS group showed higher freezing behavior than the control group in all 3 retention tests (Figure 4). There were significant main effects for group (*F*_1,20_ = 10.8, *p* = .003) and delay (*F*_2,44_ = 7.58, *p* = .001). No significant interaction between group and delay was observed (*F*_2,44_ = 1.42, *p* = .21). Post hoc tests revealed that the MS group had a higher average freezing time than the control group after 24 hours (*t*_13.85_ = 2.45, *p* = .04), a 9-day delay (*t*_15.89_ = 2.39, *p* = .04), and a 16-day delay (*t*_13.38_ = 2.43, *p* = .04). Some individuals in the MS group showed little or no freezing when used as controls in the retention test with a 16-day delay (day 31).

### Total Brain Volume Changes

**Figure 5:**
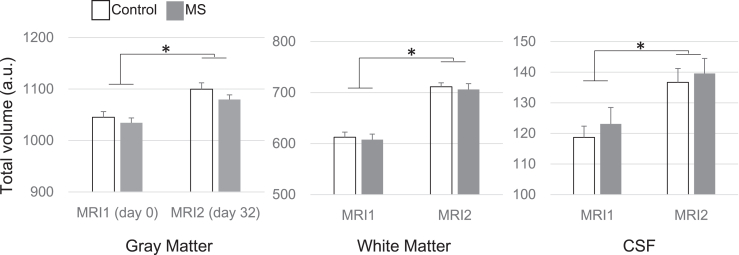
Total volumes of brain tissues before and after treatments. There were no group differences in total gray matter, white matter, or CSF volumes on MRI 1 or 2. Brain volume in both groups were increased at MRI 2 relative to MRI 1. Error bars show standard deviations. ∗Significant group difference, *p* < .001. a.u., arbitrary unit; CSF, cerebrospinal fluid; MRI, magnetic resonance imaging; MS, multiple stress.

Before (MRI 1) and after (MRI 2) MS treatment with the series of behavioral tests, no significant group differences were observed in the total GMV, WM volume, or total CSF. In MRI 2 on day 32, both groups showed greater volumes of GM, WM, and CSF than that before treatment as seen in MRI 1 (Figure 5).

### Group Comparisons for rGMV Changes

**Figure 6:**
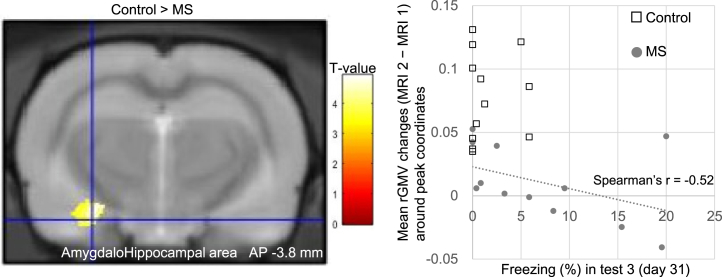
Reduced rGMV with MS associated with freezing behavior. Lower amygdalohippocampal volume in the MS group than in the control group was detected in longitudinal analyses. Familywise error–corrected *p* < .05 at the cluster level; uncorrected *p* < .001 at the voxel level. Percentage freezing time in retention test 3 of fear conditioning was negatively associated with rGMV changes in the left amygdalohippocampal area in the MS group. AP, anterior-posterior; MRI, magnetic resonance imaging; MS, multiple stress; rGMV, regional gray matter volume.

In the results of the VBM analysis for each time point before or after MS treatment, no statistically significant group differences in rGMV were detected for either MRI 1 or MRI 2. Longitudinal VBM analyses revealed reduced rGMV in the anterolateral part of the amygdalohippocampal area in the MS group compared with the control group (Figure 6 and Table 1). No greater rGMV was observed in the MS group than in the control group. Moreover, greater freezing responses in fear condition retention test 3 on day 31 in the MS group were associated with reduced or less increased amygdalohippocampal area (Spearman’s *r* = −0.52, *p* = .04).

**Table 1:** Brain Region Showing Smaller Volume Changes in the Multiple Stress Group Than in the Control Group

Brain Region	Number of Voxels	Peak *t* Value	Peak *p* Value	Peak Position		
				ML	AP	DV
Amygdalohippocampal Area	740	*t*_22_ = 4.92	4.00	−3.8	−3.8	8.2

ML, AP, and DV show the peak position relative to bregma in the stereotaxic space.

AP, anterior-posterior; DV, dorsal-ventral; ML, medial-lateral.

## Discussion

Our longitudinal behavioral and MRI studies revealed that severe MS could induce persistent anxiety-like behaviors and structural brain changes in animals. Reduced and/or decreased volume of the amygdalohippocampal area due to MS may be associated with enhanced freezing responses. These individual variabilities in the relationship between anxiety-like behaviors and brain volume reduction may provide new insights into the neurobiological mechanisms of PTSD.

The MS group consistently showed enhanced exploratory behaviors in the open field test and longer freezing times in the 3 retention tests of fear conditioning than the control group. These long-lasting fear-/anxiety-like behaviors suggest that multiple physical stress treatments can induce a PTSD-like phenotype (7). In addition, individual variability in freezing time in the last retention test in the MS group implied that susceptible and resilient animals responded similarly to trauma-exposed humans.

Young adult rats in both groups showed increased total brain volume at 32 days, and no group differences were observed. While the control group showed a slightly increased volume in the amygdalohippocampal area, a reduction and/or less increase in amygdalohippocampal area volume was observed in the MS group. Smaller volumes in the amygdalohippocampal area after MS could be related to prolonged anxiety-like behavior, and stress-induced neural and behavioral changes could show individual variability. Furthermore, changes in the amygdala-hippocampus circuit could be involved in prolonged contextual fear conditioning in the MS group (18). A coordinate-based (not region of interest–based) meta-analysis revealed a volume reduction in the left amygdala and hippocampus of patients with PTSD (19). These results suggest that a smaller amygdalohippocampal area triggered by severe stress in animals may be a sign of PTSD.

The posterior nucleus of the amygdala (amygdalohippocampal area) in rats is associated with fear-/anxiety-related regions, such as the basolateral amygdala, ventral subiculum, dorsal raphe nucleus, and infralimbic cortex (20,21). In mice, the amygdalohippocampal area is involved in aggressive (22,23) and anxiety-like behavior for less time in the central area in the open field test (24). Vulnerability of the amygdalohippocampal area has been identified during seizures in rodents (21). In humans, PTSD severity has been associated with a smaller volume in the amygdala-hippocampal transition area, which is adjacent to the amygdalohippocampal area (25). Structural and functional degeneration in and vulnerability of the amygdalohippocampal area may be associated with persistent fear- and anxiety-like behaviors.

This study had several limitations. We used only adult male Wistar rats. A single rodent model cannot perfectly capture the complexity of PTSD in humans because a variety of traumas can trigger PTSD. Although individual animal models can replicate the lack of coping and ineffective adaptation to stress, they do not necessarily represent the coverage of human PTSD variability (4). Sex differences in PTSD have been shown at epidemiological, neurobiological, and hormonal levels (26), especially that differences in the balance between activating hippocampal and amygdala circuits during contextual fear conditioning may be associated with the increased PTSD risk among females (18). Additional studies including female animals are required to understand PTSD etiology. In addition, we used only the open field test, but another anxiety measure would be necessary to confirm the effects of MS on anxiety-related behavior.

### Conclusions

This longitudinal study combining MS and in vivo MRI revealed the long-lasting effects of traumatic experiences on the brain and behavior of adult male rats. Individual variability in the amygdalohippocampal area may be related to an enhanced freezing response after severe stress. The neurobiological mechanisms of PTSD-like behavior observed in this study may contribute to the development of PTSD treatment. Additional studies are required to examine the effects of early-life stress, sex, and strain on the PTSD-like phenotype and brain.

## Supplementary Material

Key Resources Table
